# Crystal plasticity simulations with representative volume element of as-build laser powder bed fusion materials

**DOI:** 10.1038/s41598-023-47651-2

**Published:** 2023-11-21

**Authors:** Dmitry S. Bulgarevich, Sukeharu Nomoto, Makoto Watanabe, Masahiko Demura

**Affiliations:** National Institute for Materials Science, 1-2-1 Sengen, Tsukuba, Ibaraki 305-0047 Japan

**Keywords:** Metals and alloys, Computational methods

## Abstract

Additive manufacturing of as-build metal materials with laser powder bed fusion typically leads to the formations of various chemical phases and their corresponding microstructure types. Such microstructures have very complex shape and size anisotropic distributions due to the history of the laser heat gradients and scanning patterns. With higher complexity compared to the post-heat-treated materials, the synthetic volume reconstruction of as-build materials for accurate modelling of their mechanical properties is a serious challenge. Here, we present an example of complete workflow pipeline for such nontrivial task. It takes into account the statistical distributions of microstructures: object sizes for each phase, several shape parameters for each microstructure type, and their morphological and crystallographic orientations. In principle, each step in the pipeline, including the parameters in the crystal plasticity model, can be fine-tuned to achieve suitable correspondence between experimental and synthetic microstructures as well as between experimental stress–strain curves and simulated results. To our best knowledge, this work represents an example of the most challenging synthetic volume reconstruction for as-build additive manufacturing materials to date.

## Introduction

The laser powder bed fusion (LPBF) belongs to a family of additive manufacturing (AM) technique which enables to form layer by layer a more or less complex 3D parts. For metal materials, it usually involves the rapid repeating melting and solidification of powder granules. Depending on device settings such as laser power, its focus diameter, scan speed, scan interval, scan pattern, powder bed layer thickness and support shape as well as powder properties such as particle size, powder flowability, powder bed density, and its physico-chemical properties, the very complex microstructure patterns are formed in as-build materials. Such microstructure could evolve further during the heat treatment scheduling, but it is desirable to understand the microstructure-property relationships and to optimize the as-build material microstructure in order to minimize the production energy consumption for any practical applications. In this respect, the computer simulation of the mechanical properties of as-build LPBF materials is an important topic.

In previous reports, the total representative volume element (RVE) for a finite element method (FEM) simulations with a rate-independent single-crystal plasticity model was reconstructed from optical microscopy imaging of selective laser melting (SLM) 316 L material (from pre-alloyed stainless steel powder) by using the grain-scale semi-circle and columnar Voronoi tessellations^1^. A good agreement was observed between experimental and simulation results by calibrating of elastic and plastic material constants. However, the grain sizes and shapes as well as their morphological and crystallographic orientations did not correspond directly to the experimental data. This shortcoming was recently addressed in similar work on stainless steel 316L and titanium alloy Ti-6Al-4 V materials^2^. In this regard, the most realistic RVE reconstruction of 316L stainless steel sample (wire + arc AM method) was obtained by using of 3D anisotropic Voronoi algorithm. The RVE with columnar grains, which are gradually elongated and oriented along the building direction, were also used with CP FEM simulations on stainless steel 316L and aluminum AA6016 samples. However, only visual correlations of RVEs with experiment were discussed^3–5^. In addition, the case study for single-phase austenitic high-manganese steel fabricated by LPBF was reported. The RVEs were simulated with a kinetic Monte Carlo (MC) or MC Potts models coupled with physics-based crystal plasticity (CP) modeling^6,7^. Though, no detailed comparison with experimental material texture and mechanical data were given. Sometimes, the manual sketching of grain boundaries from EBSD 2D IPF maps is used with following extrusion along the Z-direction to make a 3D model with random crystallographic texture for CP FEM simulations^8^. The most realistic and recently published RVE generations known to us to date are by statistical representations of grain sizes, shapes, and orientation distributions obtained by EBSD for Hastelloy-X fabricated with SLM technique^9^, for Ti-6Al-4 V alloys with electron beam melting powder-bed fusion (EBM-PBF)^10^ or LPBF^11^ and for high-manganese steel (HMnS) processed by LPBF^12,13^. The latter works on HMnS should be especially pointed out since they are most related to the current one in terms of RVE reconstruction approach and simulation methods. Nevertheless, besides the obvious use of different material and LPBF settings in our work, we believe that our microstructure complexity is more demanding and requires additional object shape and spatial distribution tools to get the reasonable RVE reconstruction as it will be discussed below. Other works on AM with CP simulations with less demanding material textures were also published^14–16^.

Therefore, there are several strategies to generate RVEs for subsequent CP simulations: (1) additional 2D/3D computer simulations of LPBF process to get image data, (2) mimicking of microscopy 2D image data, (3) one-to-one reconstruction of RVE from experimental 3D sliced imaging, and (4) statistical reconstruction from experimental 2D or 3D image data. Here it should be pointed out that (1) requires additional verification with experimental data, (2) is less accurate among all and apparatuses for (3) are not available in most labs. Moreover, the routes (1)–(3) are sample specific and not flexible in terms of RVE use in CP simulations. Consequently, we focused our studies on (4) with 2D image data for RVE reconstruction as the most practical approach which is equally applicable with 3D image data if available.

In this regard, our work is on realistic statistical reconstruction of RVE from experimental microscopy data of a very complex microstructure pattern in as-build LPBF sample. In this sense, the RVE is not a visually identical copy of experimental sample, but a statistical one in terms of microstructure properties. This allows to be flexible in simulating of different AM samples and to design the optimum microstructure with desired mechanical properties of AM materials. Here it should be also stressed that our RVE is the most accurate example reported to date in terms of combined morphological, textural and spatial distributions of several grain types in single RVE. This is apparent if to compare our experimental and RVE images discussed below with referred ones above. We also used the different RVE reconstruction pipeline and CP simulation tools or model compared to recently published ones^9,12,13^.

## Material and methods

**Figure 1:**
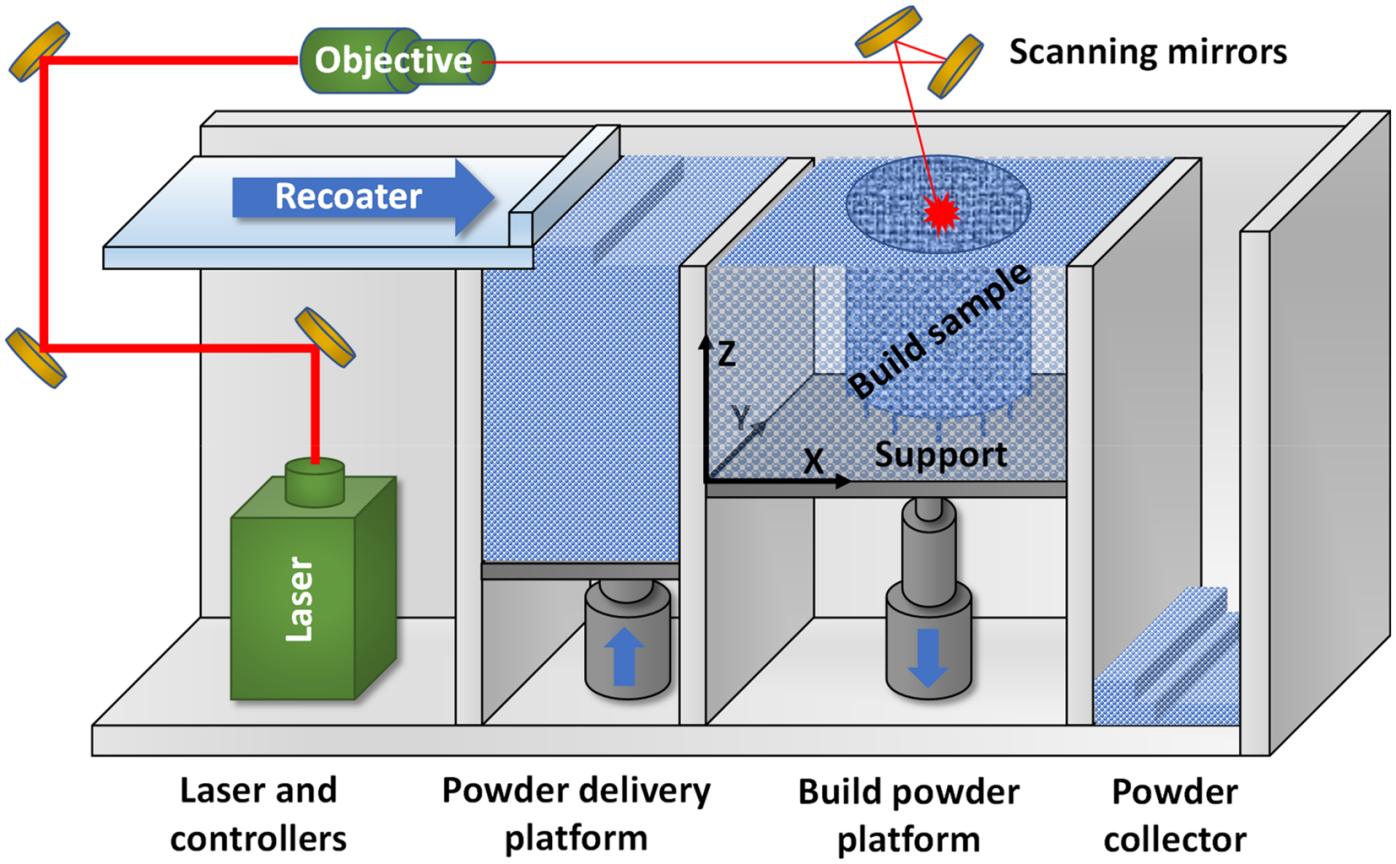
Schematic of the LPBF process.

The Inconel 738LC (IN738LC) cylindrical specimens were fabricated on SLM280HL from SLM Solutions Group AG (Lübeck) with 300 W laser power at 1064 nm wavelength, 80 μm spot diameter (Gaussian profile), 1100 mm/sec scan velocity, and 100 μm scan pitch (see Fig. 1). The stripe scan pattern was applied with 100 μm scan pitch, and the scanning direction was rotated 90° every layer. The INC738LC powder (AMPERPRINT 0151; Höganäs AB), a Ni-based superalloy with a particle size of 15–45 μm, was used with chemical composition summarized in Table 1. The powder layer thickness was about 30 µm. The cylindrical samples with the diameter of 10 mm and the height of 8 mm were built with the support length of 3 mm from the stainless steel base plate. The LPBF samples were analyzed in XY and XZ planes by a scanning electron microscope (SEM, JSM-7200F; JEOL Corp.) with electron backscatter diffraction (EBSD) as well as with X-ray computed tomography (X-ray CT, SMX-160CTS; Shimadzu Corp.).

**Table 1:** Inconel 738LC powder composition.

Element	Ni	Al	B	C	Co	Cr	Mo	Nb	Ta	Ti	W	Zr	O
Composition	Bal	3.5	0.007	0.1	8.5	15.9	1.7	0.88	1.8	3.5	2.5	0.024	0.017

The data pipeline and simulations were mainly conducted with Dream3D and DAMASK open source packages^17,18^ on two-CPU 6128 Opteron Workstation with 128 GB RAM. For comparison with Dream3D reconstructed RVE, the RVE with γ-equiaxed/columnar solidification microstructure was also simulated with LPBF thermal conditions by using non-equilibrium multi-phase field method (MPFM) based on a finite interface dissipation model and coupled with the Calculation of Phase Diagram (CALPHAD) database for a multicomponent INC738LC alloy with Ni(Bal.)-Al-Co-Cr-Mo-Ta-Ti-W-C principal components^19^. The RVEs reconstructed with Dream3D 6.5.150^20^ and color maps from simulations with DAMASK 2.03^21^ in all Figures below were visualized with ParaView 5.9.0-RC2^22^.

## Results and discussion

**Figure 2:**
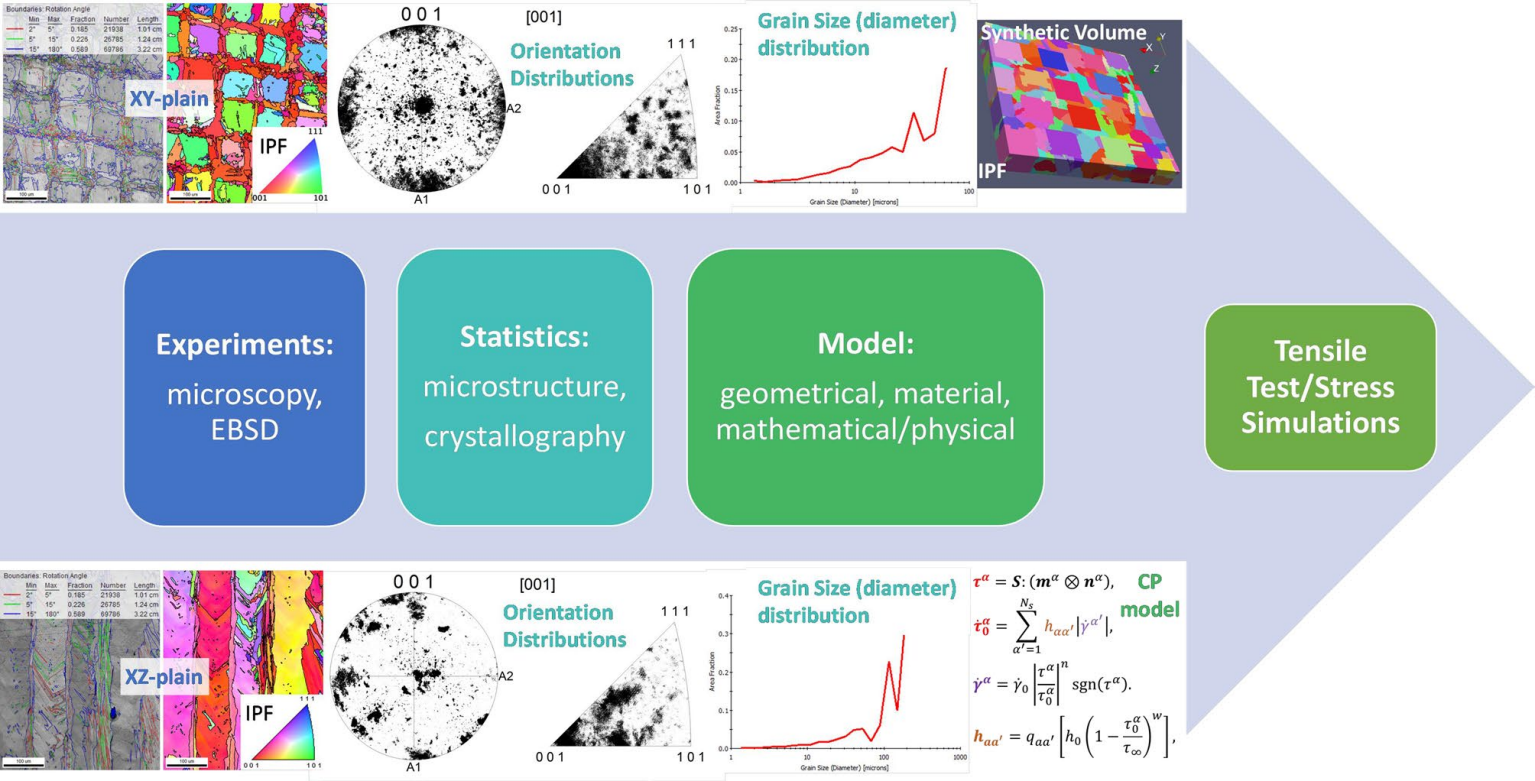
Data flow pipeline for tensile test simulation of metal materials.

Figure 2 shows the general outline of data pipeline to simulate the tensile test of a metal material. It starts from collecting of the microscopy data in digital form with SEM, EBSD and X-ray CT techniques from LPBF samples, then it continues by extracting of the relevant statistical information from observed microstructures, it is following by RVE reconstruction and preparation of the relevant input data for CP modelling, and it is finishing by conducting of the tensile test simulations. Each step in the pipeline can be modified depending on previous input, desired microstructure type or materials parameters. As a demonstration, the detailed explanation is given below for LPBF fabricated IN738LC sample material.

**Figure 3:**
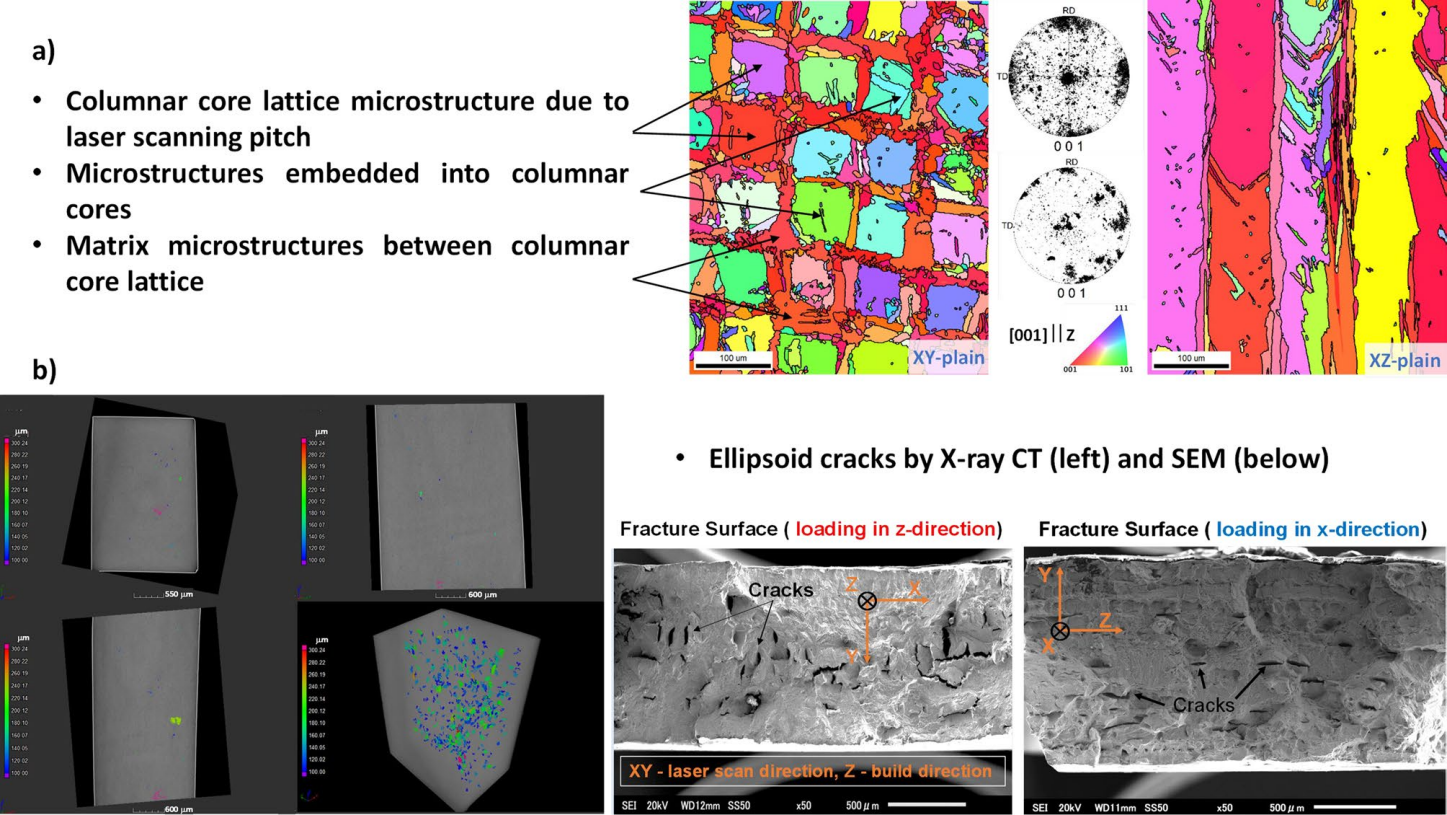
Microstructures in as-build IN738LC: (**a**) EBSD images in IPF colors and crystallographic ODF plots, (**b**) X-ray CT and SEM images.

In our case, the SEM and EBSD apparatuses can output data tables with equivalent circle diameter (ECD), minimum and maximum Feret diameters, crystallographic Euler angles for each grain and 2D images in IPF and/or grain ID colors. Here it should be stressed that RVE reconstruction is attempted from 2D sliced imaging in XY and XZ planes since there is no statistical anisotropy between XZ and YZ planes (see Fig. 3). Otherwise, a more complex RVE reconstruction will be required from 2D data^23,24^ or preferably the 3D image data should be used^11,25^. The complexity of the present task can be seen from Fig. 3a and b with listed microstructure types: the columnar core lattice microstructure due to the laser scanning pitch (Columnar Core), microstructures embedded into columnar cores (Embedded), matrix microstructures between Columnar Core lattice which are elongated on average along $x$- or $y$-axis (Matrix-$\parallel$ + Matrix-$\perp$, respectively), and cracks with different orientations along $x$-axis (Crack-$\parallel$ and Crack-$\perp$). Due to very rapid solidification with LPBF process and used microscopy magnification, no $\mathrm{\gamma {\prime}}$ microstructural objects/phases could be distinguished in EBSD data/images. This is also consistent with our 2D phase field simulations^19^.

### RVE reconstruction

To define each microstructure type from Fig. 2 for RVE reconstruction in Dream3D by using of appropriate filter list, the following information is required: (1) the volume fraction (${f}_{V}$), (2) size distribution, (3) morphology, and (4) texture of the microstructure objects. The property (2) is approximated with log-normal shape of probability density function (PDF) for the mean equivalent sphere diameter (ESD) of microstructure objects, ${PDF}_{L-N}=exp\left[-{\left(ln\left(ESD\right)-\mu \right)}^{2}/2{\sigma }^{2}\right]/x\sigma \sqrt{2\pi }$, where $\mu$ and $\sigma$ are the mean and standard deviation of the $ln\left(ESD\right)$. The ${ESD}^{\left(\mu -min\times \sigma \right)}$ and ${ESD}^{\left(\mu +max\times \sigma \right)}$ are the minimum and maximum cut offs for ${PDF}_{L-N}$. To control statistics for different sizes, the number of bins in ${PDF}_{L-N}$ is defined by the bin step size ($BSS$), $numBins=\left({e}^{\mu +\left(max\times \sigma \right)}-{e}^{\mu -\left(min\times \sigma \right)}\right)/BSS$.

The property (3) is defined by the PDF of ${\overline{\Omega } }_{3}$, ${PDF}_{B}=\left({ESD}^{\alpha -1}{\left(1-EDS\right)}^{\beta -1}\right)/B\left(\alpha ,\beta \right)$, with beta function $B\left(\alpha ,\beta \right)$ having two ${PDF}_{B}$ shape parameters $\alpha$ and $\beta$. The ${\overline{\Omega } }_{3}$ is a parameter representing the object shape and it is a function of 3-D moment invariants in second-order moment matrix that are independent of the reference frame and are useful as quantitative shape descriptors due to their invariance with respect to either similarity (translation, rotation, isotropic scaling) or affine (similarity + anisotropic scaling and shearing) coordinate transformations^26,27^. As a result, various ellipsoids, superellipsoid, and cube-octahedra shapes such as cubes, cuboids, octahedral, spheres, etc. and their anisotropic equivalents described by superellipsoid equation, ${\left|x/a\right|}^{n}+{\left|y/b\right|}^{n}+{\left|z/c\right|}^{n}\le 1$, where $b/a$ and $c/a$ are the aspect ratios, can be created with ${\overline{\Omega } }_{3}$ linked to exponent $n$ and to the clipping depth $\gamma$ for cube-octahedra. The microstructure object orientation in a morphological sense is controlled with axis orientation distribution function (ODF) that orients the three principal axes of the grains with respect to XYZ-axes of RVE. Such axis OFD can be controlled with Euler angles, weight ($w$) in units of multiples of random distribution (MRD), and $\sigma$ used for blurring out of the chosen orientation. If some symmetry in microstructure object distribution is present, then radial distribution function (RDF) can be introduced to reflect it in RVE. Finally, the property (3) for each microstructure type is introduced by crystal lattice type and ODF for [001], [011], and [111] crystal axes. Then, packing of the microstructure objects with texture matching is attempted in a Monte Carlo fashion to optimize the space filling and local/neighborhood feature arrangement.

To keep reasonable CP simulation time and consumption of PC resources, the RVE was restricted to 128 × 128 × 16 pixels which correspond to 400 × 400 × 50 μm^3^. The lower pixel count in Z-axis direction was due to inherent periodic boundary conditions used in CP simulation with Fourier approximation of the deformation gradient in Spectral Solver.

**Figure 4:**
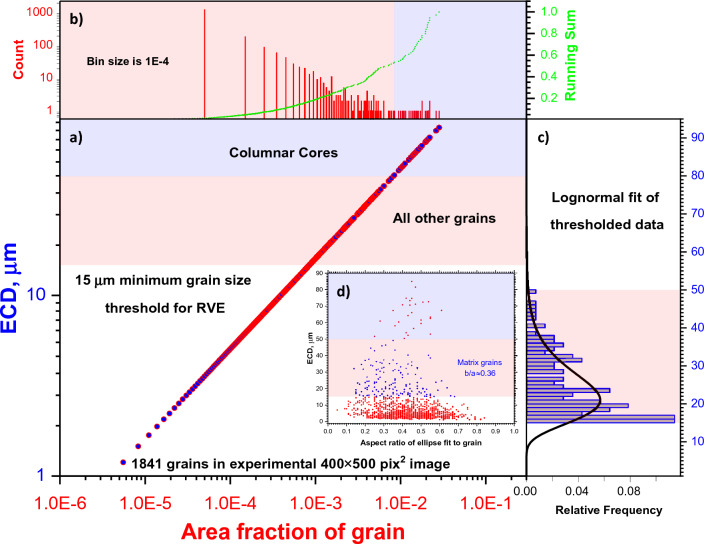
The outline of EBSD data statistical analysis for XY-plane image from Fig. 3. (**a**) The central panel plot demonstrates the thresholding of the grain size data in terms of ECD on Columnar Core and other microstructures. (**b**) The top panel is used for estimation of Columnar Core ${{\varvec{f}}}_{{\varvec{V}}}$ based on running sum of grain area fractions. (**c**) The right panel plot illustrates the lognormal fit of thresholded ECD distribution for evaluation of ${\varvec{\mu}}$ and ${\varvec{\sigma}}$ parameters in ${{\varvec{P}}{\varvec{D}}{\varvec{F}}}_{{\varvec{L}}-{\varvec{N}}}$. (**d**) The insert in central panel plot is used to estimate the ${\varvec{b}}/{\varvec{a}}$ parameter ratio for thresholded ECD distribution. See text for more details.

They take care to recreate the columnar microstructure along Z-axis (see Fig. 3a). Such spatial resolution also limits the minimum ESD size of the microstructure object in RVE. The microstructures seen in Fig. 3a were introduced into RVE with parameters listed in Table 2. The ${f}_{V}$, ${PDF}_{L-N}$, and aspect ratios were derived from statistical analysis of EBSD data from Fig. 3. As an example, see Fig. 4 for analysis of XY-plane image. The initially derived parameters were taken as a guideline and reasonably tuned to keep manageable number of grains for DAMASK simulation with RVE and to have visual correspondence with experimental image data. The crystal ODFs for Columnar Core, Matrix-$\parallel$ and Matrix-$\perp$ microstructures were fed directly from EBSD data into Dream3D. The shape types, axis ODFs, and RDF were defined/approximated from visual analysis of images in Fig. 3. Additional details on values of key parameters in Table 2 are given below.

**Table 2:** Parameters to define the microstructures in Dream3D for RVE reconstruction from Fig. 3 and EBSD data tables.

Microstructure	${f}_{V}$	Shape type	${PDF}_{L-N}$					${PDF}_{B}$		Aspect ratio		Axis ODF			RDF	Crystal ODF		
			$\mu$	$\sigma$	$BSS$	$min$	$max$	$\alpha$	$\upbeta$	$b/a$	$c/a$	Euler angles	$\sigma$	$w$		Euler angles	$\sigma$	$w$
Columnar core	0.626	Cube octahedron	4.45	10^–3^	1	0.1	0.1	140	42	1	1	(0,0,0)	1	500,000	Square lattice	Directly sampled from EBSD data table		
Matrix-$\parallel$	0.134	Cube octahedron	3.5	0.5	40	1	2	140	42	0.36	0.2	(0,0,0)	1	50,000	–	Directly sampled from EBSD data table		
Matrix-$\perp$												(90,0,0)						
Embedded	0.104	Supper ellipsoid	3.8	0.1	1	5	5	10	1.7	1	0.05	(270,90,90)	1	5000	–	(270,90,90)	1	5000
Crack-$\parallel$	5.6 × 10^–4^	Supper ellipsoid	3.8	0.1	1	5	5	10	1.7	1	0.05	(270,90,90)	1	50,000	–	(0,0,0)	5	1
Crack-$\perp$												(0,90,270)

**Figure 5:**
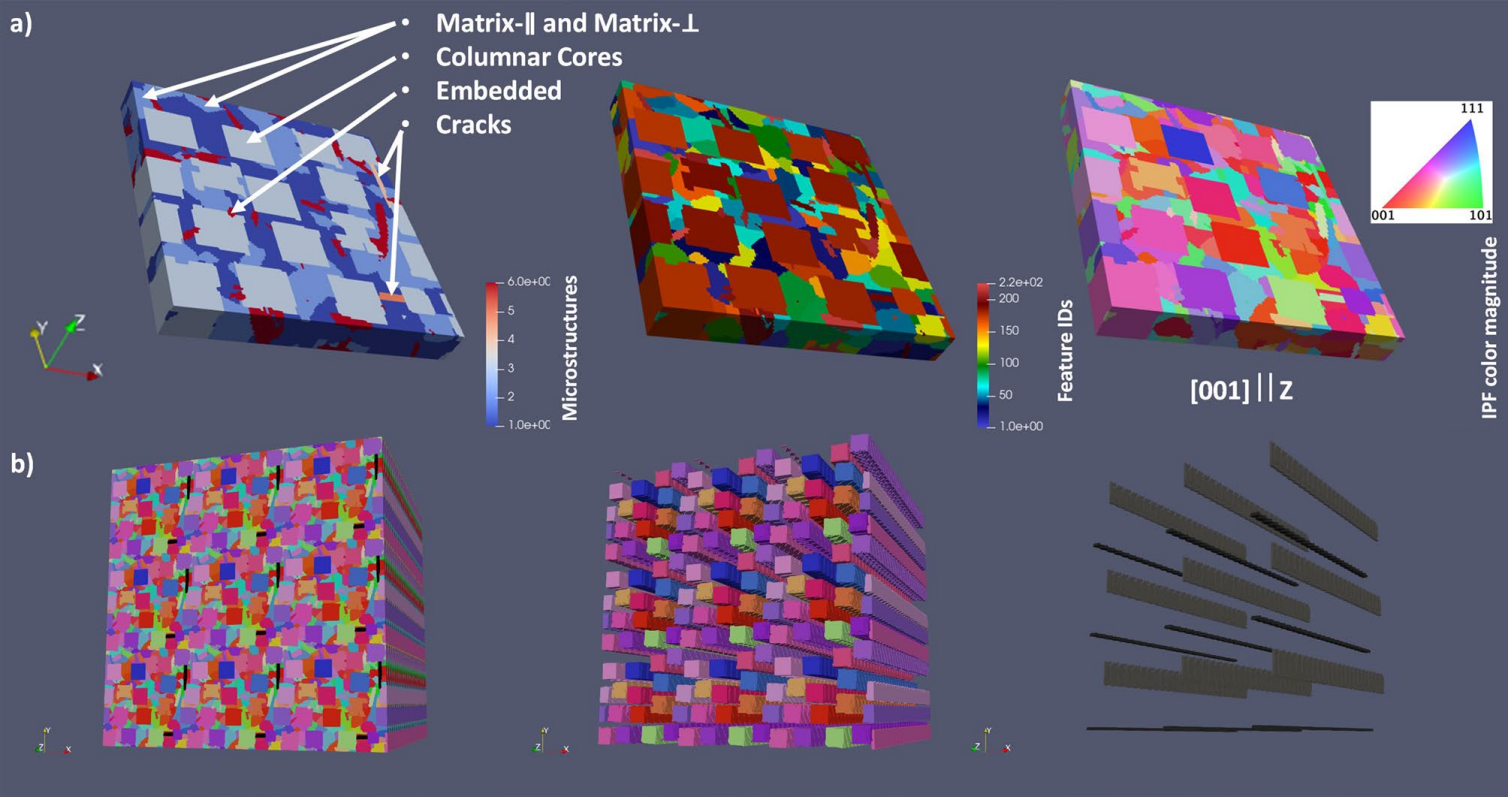
The reconstructed synthetic volume: (**a**) RVE in three color schemes: microstructure-wise, grain-wise, and grain crystallography-wise (except cracks); (**b**) the cloned RVE along $x$, $y$, and $z$- axes: all microstructures, thresholded columnar ones, and cracks from right to left, respectively.

We set the ECD = 15 μm as the lower bound to have more than 300 pixel per object in RVE with $ESD=2\cdot \sqrt[3]{3{V}_{c}/4\pi }\approx 26 \mu m$, where ${V}_{c}=\pi \cdot {\left(ECD/2\right)}^{2}\cdot z$ and $z$ is a column length in RVE. This also helps to avoid the very sharp object edges which may prevent the convergence in CP simulations due to very high stress localizations. From EBSD data tables, the ECD data were thresholded into Columnar Core and (Matrix-$\parallel$ + Matrix-$\perp$ + Embedded) objects (see Fig. 4). After threshold, the estimated mean value of $\overline{ECD }$≅24 μm for (Matrix-$\parallel$ + Matrix-$\perp$) and Embedded objects was used. For Columnar Core objects, the $\overline{ECD }$≅64 μm, however, the best correspondence with Fig. 3a was achieved with $\overline{ECD }$≅46 μm after object packings into RVE with square lattice RDF. The ${f}_{V}$ values for Columnar Core and (Matrix-$\parallel$ + Matrix-$\perp$ + Embedded) microstructures were approximated from corresponding thresholded areas as described above with Fig. 4. The separate ${f}_{V}$ values for (Matrix-$\parallel$ = Matrix-$\perp$) and Embedded microstructures were arbitrary set to get reasonable visual correspondence with Fig. 3a. The ${f}_{V}$, dimensions, and aspect ratios for cracks were taken from X-ray CT and SEM data. As a result, the statistically reconstructed RVE is shown in Fig. 5a. To our knowledge, such realistic digital twinning of as-build LPBF sample is reported for the first time. The periodically enlarged RVE is also displayed in Fig. 5b. It has similar columnar structure with some irregularities as in experimental one.

**Figure 6:**
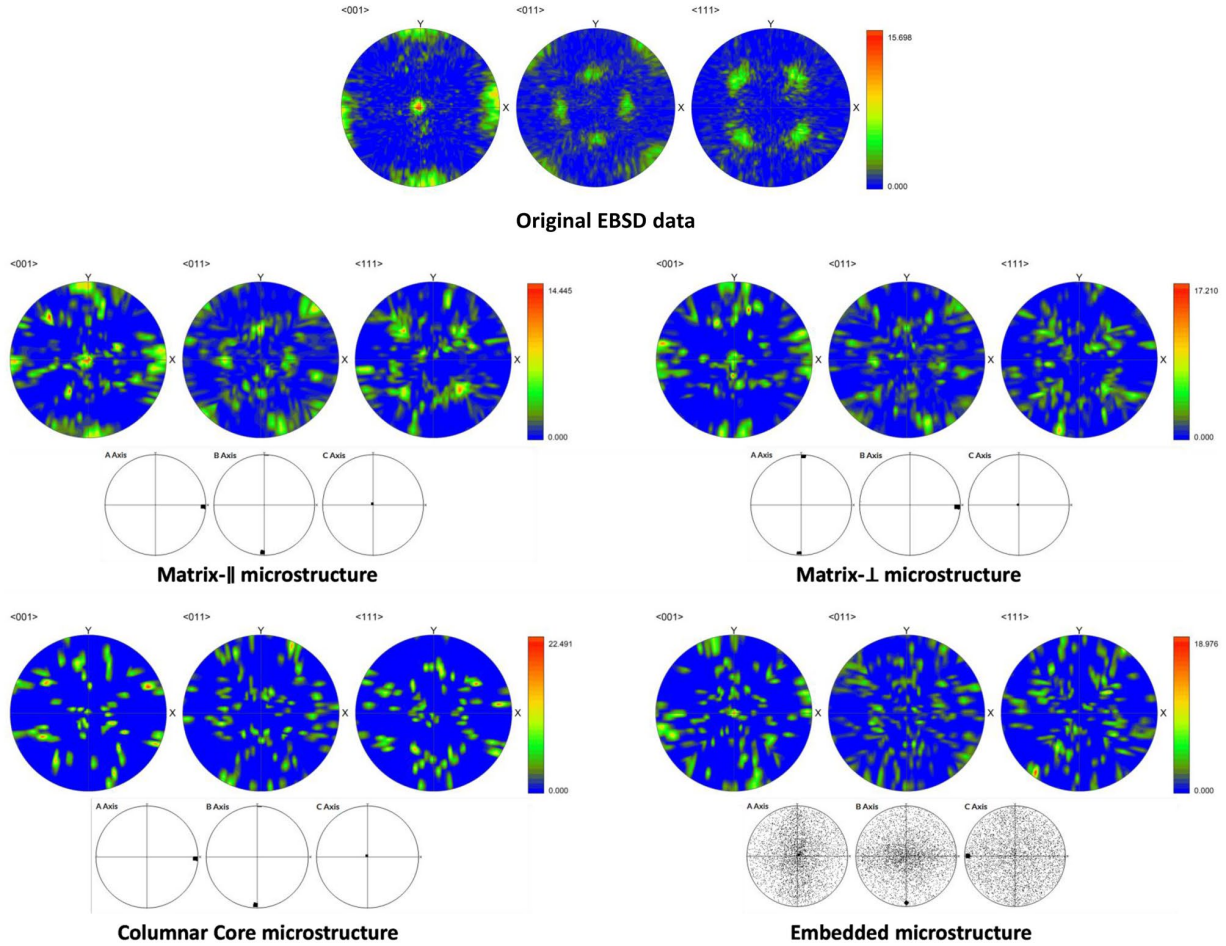
Lambert pole (color) and axes ODF figures for relevant microstructure in RVE.

In addition, the Lambert pole and axes ODF figures for relevant microstructures are demonstrated in Fig. 6. The corresponding axis and crystal ODF parameters are listed in Table 2. The good correspondence between original/experimental and reconstructed Lambert pole figures is especially pronounced for Matrix-$\parallel$ and Matrix-$\perp$ objects due to direct sampling from experimental EBSD data and their large number in reconstructed RVE.

### CP simulation

On the next step, the RVE geometry file is passed to the DAMASK Spectral Solver^18^ to simulate the tensile test results. It uses the fast Fourier transform to solve numerically the certain differential Eq. (1):

1 $$\underset{x}{\mathrm{min}}\mathcal{W}\Rightarrow \mathrm{Div}P\left(x\right)={\mathcal{F}}^{-1}\left[P\left(\mathrm{k}\right)i\mathrm{k}\right]=0,$$

with values defined in Eqs. (2), (3), (4), (5) and (6).

2 $$P\left(x\right)=\frac{\delta \mathcal{W}}{\delta F}\left(x,F,\dots \right),$$

3 $$F=\overline{F }+\widetilde{F},$$

4 $$\chi \left(x\right)=\overline{F }x+\widetilde{w}\left(x\right),$$

5 $$\widetilde{F}=\frac{\partial \widetilde{w}}{\partial x}=\widetilde{w}\otimes \nabla =\mathrm{Grad}\widetilde{w},$$

6 $$\chi \left(x\right):x\in {\mathcal{B}}_{0}\to y\in \mathcal{B}.$$

where $\mathcal{W}$ is the strain energy density, $\chi \left(x\right):x\in {\mathcal{B}}_{0}\to y\in \mathcal{B}$ $x$ is the deformation mapping of the points in reference configuration ${\mathcal{B}}_{0}$ to points $y$ in current one $\mathcal{B}$, $P\left(x\right)$ and $P\left(\mathrm{k}\right)$ are the first Piola–Kirchhoff stress responses in real and Fourier space, ${\mathcal{F}}^{-1}$ is the inverse Fourier transform, $\mathrm{k}$ is the frequency vector in Fourier space, $F$ is the total deformation gradient tensor with a spatially homogeneous part $\overline{F }$ and locally fluctuating displacement $\widetilde{F}$, the $\chi \left(x\right)$ is the deformation, and $\widetilde{{\varvec{w}}}$ is the displacement fluctuation field that ${\widetilde{{\varvec{w}}}}^{-}={\widetilde{{\varvec{w}}}}^{+}$ on corresponding surfaces $\partial {\mathcal{B}}^{-}$ and $\partial {\mathcal{B}}^{+}$. In principle, the spectral method should provide the fastest solution with excellent error properties^28^.

In our simulations, we used the phenomenological/empirical model for plasticity which postulates an internal deformation resistance and a power-law relation between driving force and deformation rate^29^:

7 $${\tau }^{\alpha }=S:\left({m}^{\alpha }\otimes {n}^{\alpha }\right),$$

8 $${\dot{\tau }}_{0}^{\alpha }={\sum }_{{\alpha }^{\mathrm{^{\prime}}}=1}^{{N}_{s}}{h}_{{\alpha \alpha }^{\mathrm{^{\prime}}}}\left|{\dot{\gamma }}^{{\alpha }^{\mathrm{^{\prime}}}}\right|,$$

9 $${h}_{{aa}^{\prime}}={q}_{{aa}^{\prime}}\left[{h}_{0}{\left(1-\frac{{\tau }_{0}^{\alpha }}{{\tau }_{\infty }}\right)}^{a}\right],$$

10 $${\dot{\gamma }}^{\alpha }={\dot{\gamma }}_{0}{\left|\frac{{\tau }^{\alpha }}{{\tau }_{0}^{\alpha }}\right|}^{n}{\text{sgn}}\left({\tau }^{\alpha }\right),$$

with variables and parameters from Eqs. (7), (8), (9) and (10) listed in Table 3 and additional ones used in DAMASK Spectral Solver. The elastic stiffness constants from elasticity matrix were calculated from IN738LC elastic compliance constants^30^ by corresponding Eqs. from literature^31^. The mixed boundary conditions for uniaxial tension along $x$–axis (see Fig. 5) are described by Eq. (11):

11 $$\dot{\overline{F} }=\left[\begin{array}{ccc}{10}^{-3}& 0& 0\\ 0& *& 0\\ 0& 0& *\end{array}\right]{\mathrm{s}}^{-1}\quad \mathrm{ and }\quad \overline{P }= \left[\begin{array}{ccc}*& *& *\\ *& 0& *\\ *& *& 0\end{array}\right]\mathrm{Pa},$$

where $\dot{\overline{F} }$ and $\overline{P }$ are the deformation gradient rate and Piola–Kirchhoff stress tensors, respectively, with undefined components indicated by asterisks. They satisfy the mutual exclusiveness of $\dot{\overline{F} }$ and $\overline{P }$ as well as prevent rotation (undefined $\overline{P }$ off-diagonal components)^32^.

**Table 3:** Symbols, meanings and values of parameters in phenomenological model for crystal plasticity used in this study.

Value		Definition	IN738LC	Cracks: dilatational^18,33^
Variable	$\alpha$	Slip system		
	${\tau }^{\alpha }$	Resolved shear stress (analogous to Schmid’s law)		
	${m}^{\alpha }$	Vector in slip/shear direction		
	${n}^{\alpha }$	Vector along plane normal of the respective slip system		
	${\dot{\gamma }}^{\alpha }$	Plastic shear rate on each slip system		
	${\dot{\tau }}_{0}^{\alpha }$	Hardening/resistance behavior/kinetics of a slip system		
	${h}_{{aa}{\prime}}$	Hardening matrix		
Parameter	$a$	Slip hardening	0.01	2
	$n$	Strain rate sensitivity	5	20
	${q}_{{aa}{\prime}}$	Latent hardening for (coplanar) and [otherwise] slips	(1, 1), [1.4, 1.4, 1.4, 1.4]	
	${\dot{\gamma }}_{0}$	Reference shear rate, s^-1^	0.001	0.001
	${\tau }_{0}^{\alpha }$	Initial slip resistance to plastic flow, MPa	400	0.3
	${\tau }_{\infty }$	Saturation stress or resistance to plastic flow, MPa	600	0.6
	${h}_{0}$	Slip hardening, MPa	700	1
	C_11_	Elastic stiffness constants from elasticity matrix, GPa	252 (280.27)	10
	C_12_		172 (190.99)	0
	C_44_		114 (127.06)	5
	$Latt\_str$	Lattice structure	fcc	
	$Nslip$	Number of slip systems	12	

**Figure 7:**
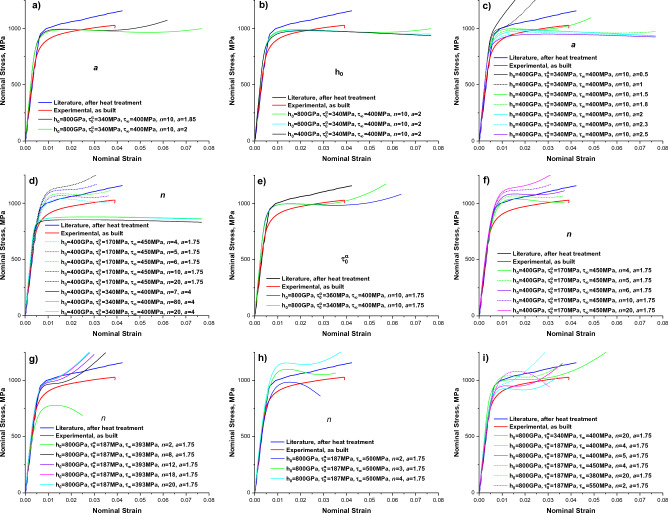
Comparison of literature^34^, experimental and simulated SSCs with various phenomenological model CP parameters. Panels (**a**–**i**) are for variation of indicated parameter by keeping all others fixed. Other parameters are the same as in Table 3.

**Figure 8:**
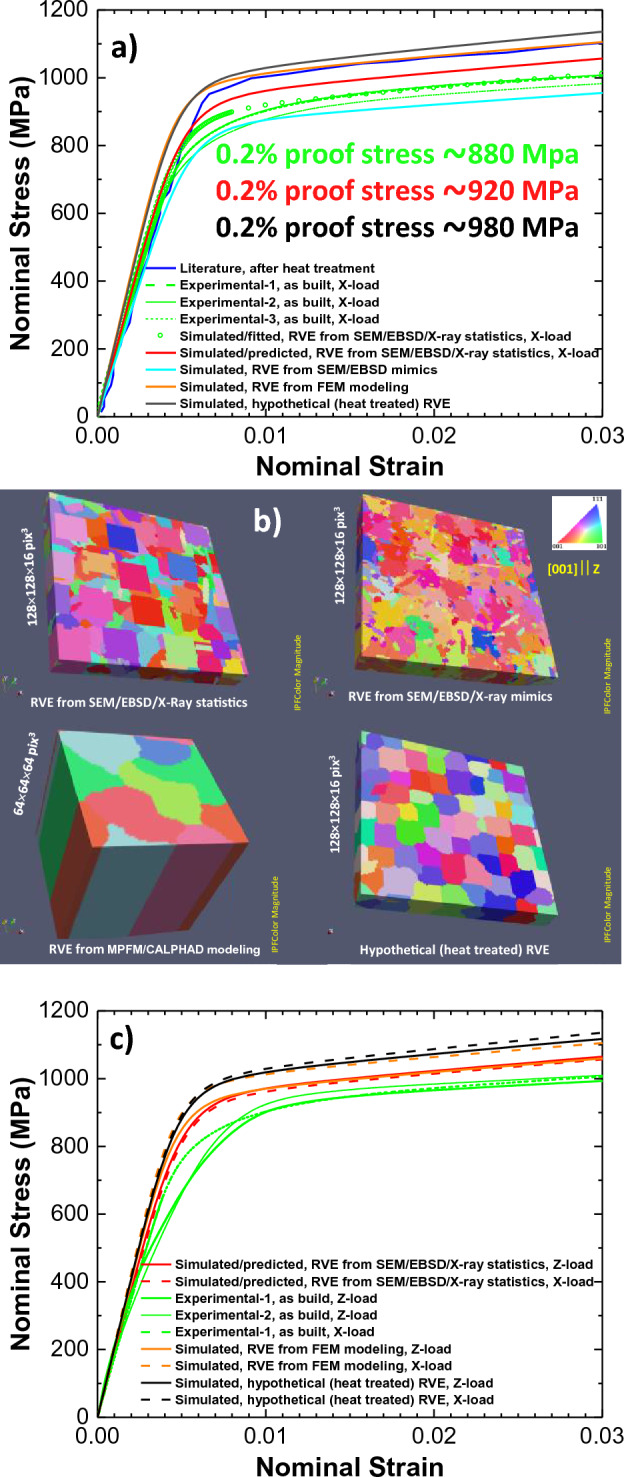
Comparison of literature and our experimental SSCs with simulated ones with uniaxial tension along $x$–axis (**a**) and $z$–axis (**c**) for different RVEs (**b**). The indicated 0.2% proof stresses are from simulated SSCs. See text for more details.

The values for IN738LC correspond to the simulated/predicted SSC shown in Fig. 8a. The elastic constants in brackets are literature data^30,31^. The reference parameters for cracks are also listed. See text for more details and Fig. 7 with additional examples.

Figure 7 shows the literature^34^, experimental, and several simulated stress–strain curves (SSCs). The simulated SSC is plotted in terms of first component of “nominal” or “engineering” (force divided by the original sectional area) stress, i.e. transpose of Piola–Kirchhoff stress tensor, $\mathrm{N}={\overline{P} }^{T}$. In this Figure, the parameters $a$, $n$, ${\dot{\gamma }}_{0}$, ${\tau }_{0}^{\alpha }$, ${\tau }_{\infty }$, and ${h}_{0}$ in phenomenological model for CP simulations were varied manually. It takes ~ 28 h per simulation and ~ 7 h for subsequent post-processing on 14-CPU cores with 2400 data points per SSC to insure the convergence. Generally, the use of converge tolerance ${\varepsilon }_{eq}\ge 1$ led to the expected simulation time with used 128 × 128 × 16 grid points in our RVE^28^. However, SSCs had different lengths due to convergence problems at high strains. Moreover, the convergence observed only for simulations without cracks (dilatational voids), i.e. by setting two corresponding grains (see Fig. 5a) in RVE with dilatational materials parameters from Table 3. The slow or no convergence behavior is expected with Spectral Method for composite materials with high phase contrasts since solution may not be smooth^18,35–39^, i.e. the convergence is not insured in such case. Note that Spectral Method does not converge at all for an infinite phase contrast due to the Gibbs phenomenon or aliasing errors in the Fourier approximation. In other words, a discontinuous function approximated by a Fourier series of trigonometric functions (as shape functions) overshoot or undershoot near the discontinuity. Then, the created oscillations do not decay with number of terms^40^.

It was supposed that simulated/predicted SSC without cracks should span between literature and experimental SSCs by resembling their shapes. Note here that literature SSC is for heat-treated SLM sample without cracks and experimental SSC is for as-build sample with cracks. To achieve this, we fitted the Experimental-1 SSC by manually adjusting of the $a$, $n$, ${\dot{\gamma }}_{0}$, ${\tau }_{0}^{\alpha }$, ${\tau }_{\infty }$, and ${h}_{0}$ parameters and by using 9/10 scaled elastic constants with RVE without cracks (see Simulated/fitted SSC in Fig. 8a). The simulation mean error $\overline{Error }$ over data points in $\mathrm{s}imulated$ (Simulated/fitted) SSC can be defined by Eq. (12):

12 $$\overline{Error }=\overline{{\left(1-{SSC}_{interpolated}^{as-build}/{SSC}_{simulated}\right)\times 100\%}}.$$

In Eq. (12), the data points in experimental $as-build$ (Experimental-1, up to ~ 3% strain in Fig. 8a) were interpolated by using of strain data points in $\mathrm{s}imulated$ SSCs. The lowest $\overline{Error }\cong 2.9\%$ was achieved with our manual fitting of CP parameters.

As it is seen from Fig. 8a, there are small vertical shifts between Experimental-1 and Experimental-2 SSCs without their shape changes including the Literature SSC. After fitting of Experimental-1 SSC, only ${\tau }_{0}^{\alpha }$ parameter was increased on 20 MPa to get the vertically-shifted Simulated/predicted SSC without cracks between Experimental-1 and Literature SSC (see red SSC in Fig. 8a and estimated $\overline{Error }$ below). The corresponding parameter set for this Simulated/predicted SSC is listed in Table 3. In principle, somewhat better fits are expected by using of appropriate machine learning and optimization algorithms^29,41,42^. Compared to SSCs in Fig. 7 with classical parabolic extrema in yielding and strain hardening regions, our experimental, simulated/fitted and simulated/predicted SSCs for IN738LC demonstrate the strain hardening with linear isotropic slip hardening process and nonlinear strain rate sensitivity, i.e. $a\approx 0$ and $n>1$, respectively (see Table 3). Again, no convergence, except for several data points in elastic part of SSC, with DAMASK CP simulations was observed with crack parameters from Table 3 or with 1/10 and 1/5 scaled IN738LC parameters (${C}_{11}$, ${C}_{11}$, ${C}_{44}$, ${\tau }_{0}^{\alpha }$, ${\tau }_{\infty }$, and ${h}_{0}$) for cracks. As a result, the effect of cracks on SSC shape cannot be clarified with current DAMASK CP simulations.

### CP simulation with different RVEs

Figure 8a displays the SSCs for different RVEs without cracks by using IN738LC CP parameters from Table 3 and boundary conditions given by Eq. (11) in comparison with literature and experimental SSCs. The corresponding RVEs are shown in Fig. 8b. If to suppose that correctly simulated SSCs should roughly span between literature (heat treated, without cracks) and our experimental (as-build, with cracks) SSCs, then simulation mean error $\overline{Error }$ over data points in $\mathrm{s}imulated$ SSC can be defined by Eq. (13):

13 $$\overline{Error }=\overline{{\left[1-\left(\frac{\left({SSC}_{interpolated}^{as-build}+{SSC}_{interpolated}^{heat-treated}\right)}{2}/{SSC}_{simulated}\right)\right]\times 100\mathrm{\%}}}.$$

In Eq. (13), the data points in experimental $as-build$ (Experimental-1, up to ~ 3% strain in Fig. 8a) and $heat-treated$ (Literature) SSCs were interpolated by using of strain data points in $\mathrm{s}imulated$ SSCs. The lowest $\overline{Error }\cong 3.7\%$ was estimated for Simulated/predicted SSC with RVE without cracks from Table 2, i.e. for RVE from SEM/EBSD/X-Ray statistical data. Then, the same CP parameter set from Table 3 was applied to simulate SSCs with different RVEs from Fig. 8b. The columnar RVE (64 × 64 × 1 pixels, 20 × 20 × 1 size, 7 equiaxed/dendric grains with random crystal ODF) obtained from quasi-equilibrium 2D MPFM/CALPHAD modeling of 20 × 20 μm^2^ area size^19^ gave much higher $\overline{Error }\cong 15.0\%$ for simulated SSC. Note that this RVE corresponds to our Matrix-type microstructures with columnar structure, but without Columnar Cores due to much smaller RVE size from 2D MPFM/CALPHAD modeling which also do not take into account the laser scanning pitch effect. The SSC with RVE, which were obtained by mimicking of experimental EBSD and crystal ODF images as well as grain distribution graphs in Dream3D to get just a visual correspondence, had $\overline{Error }\cong -6.3\%$. Finally, the hypothetically heat-treated RVE with cuboidal columnar grains from Fig. 8 produced the SSC with $\overline{Error }\cong 14.7\%$.

By using same CP parameter set from Table 3, the simulated SSCs for tested RVEs had similar shapes but differed only by the vertical shifts. This indicated that despite of the corresponding microstructure differences between RVEs, the SSCs were mainly affected by the initial slip and saturation resistance to plastic flow, i.e. by ${\tau }_{0}^{\alpha }$ and ${\tau }_{\infty }$ kinematic hardening parameters, respectively. The weak anisotropy between SSCs for uniaxial tension along with $x$– and $z$–axes in Fig. 8a and c was observed experimentally and computationally but was inconclusive in terms of vertical SSC shifts. The largest anisotropy was observed with RVE from FEM modeling having only 7 grains, which suggested that the major responsible factor was not columnar morphology, but rather particular crystal orientation.

**Figure 9:**
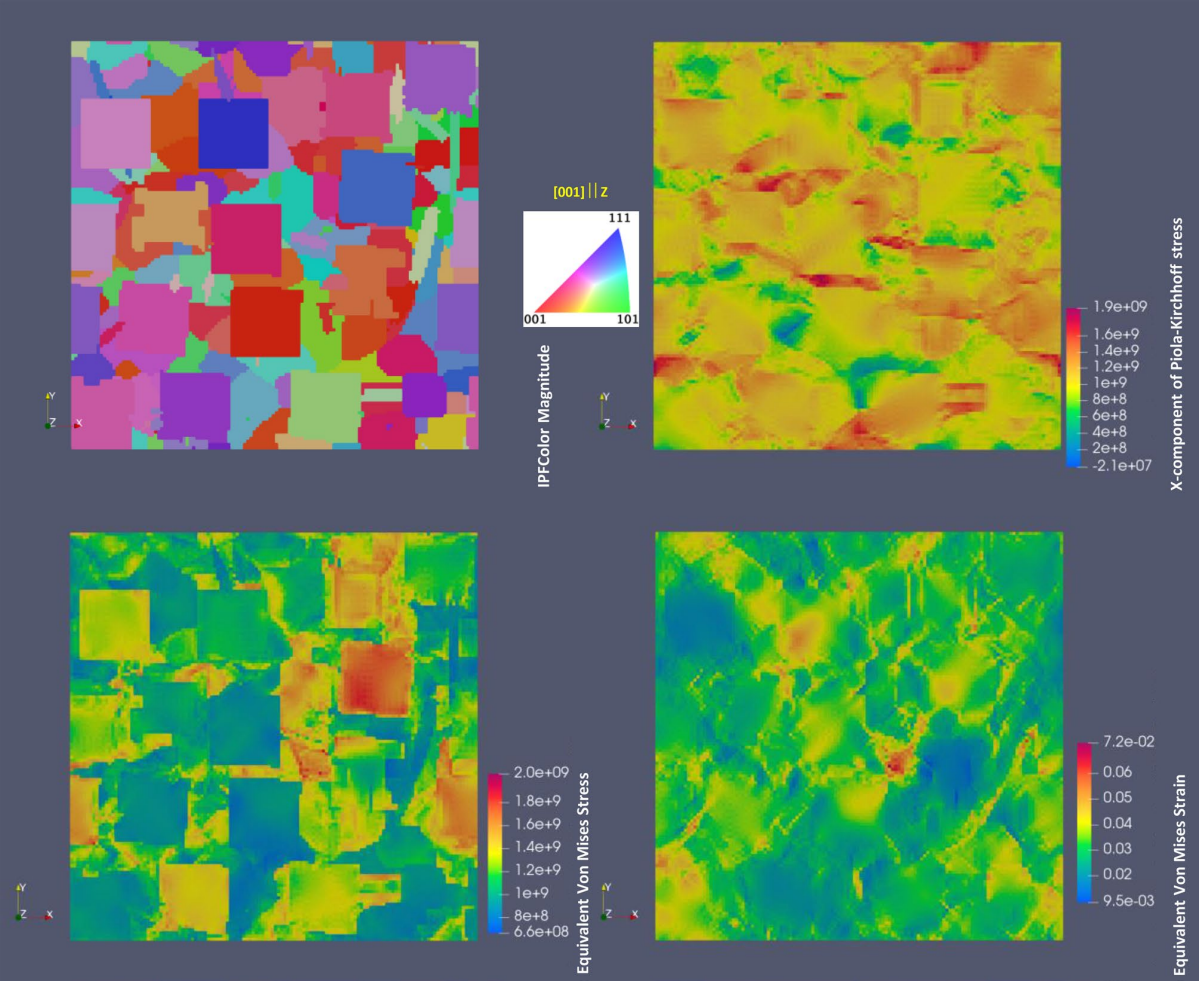
Accumulated stress and strain distribution maps for IN738LC RVE with parameters for CP from Table 3 at ~ 0.03 nominal strain.

**Figure 10:**
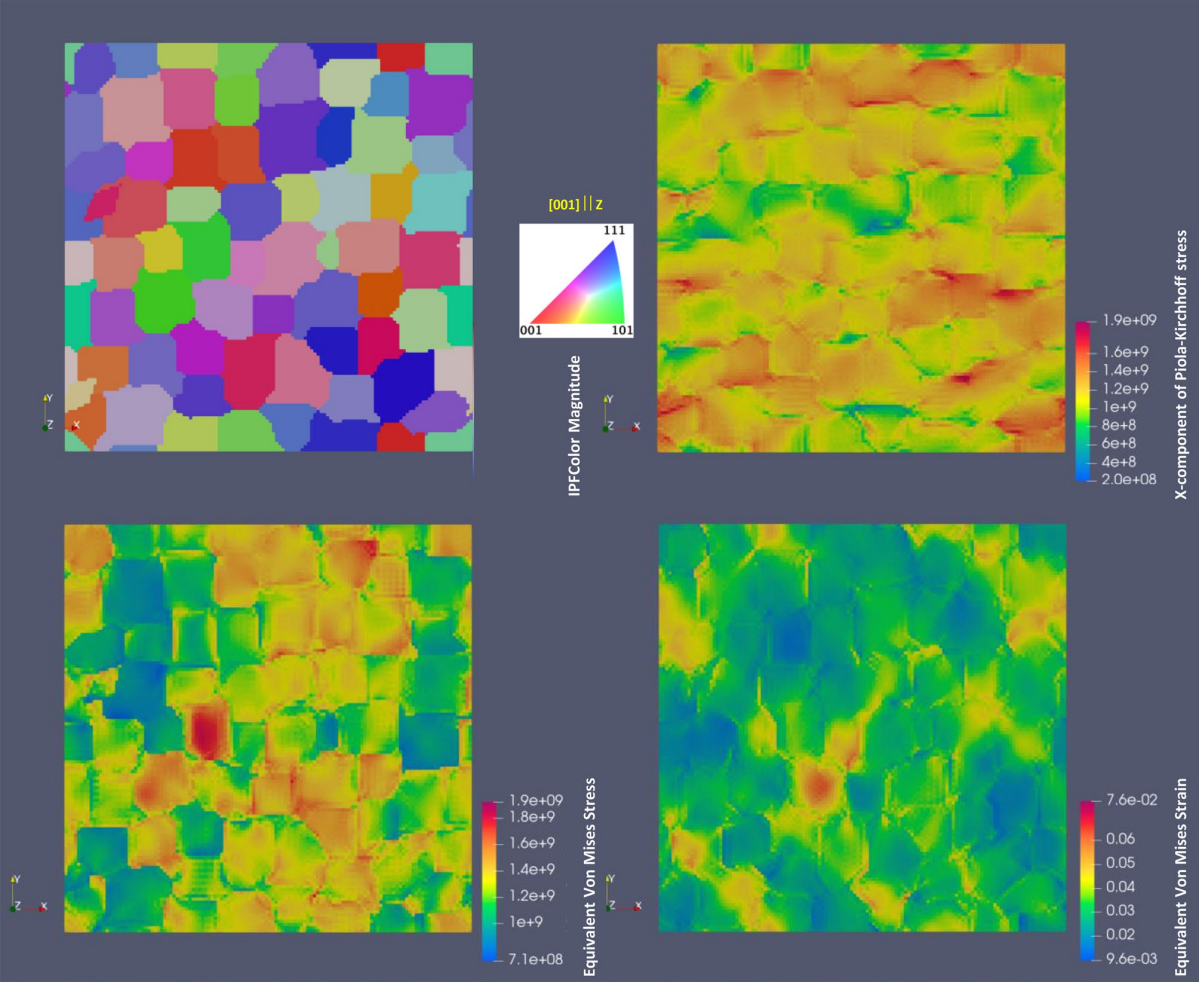
Accumulated stress and strain distribution maps for hypothetically heat treated RVE of IN738LC with cuboidal grains at ~ 0.03 nominal strain with parameters for CP from Table 3.

**Figure 11:**
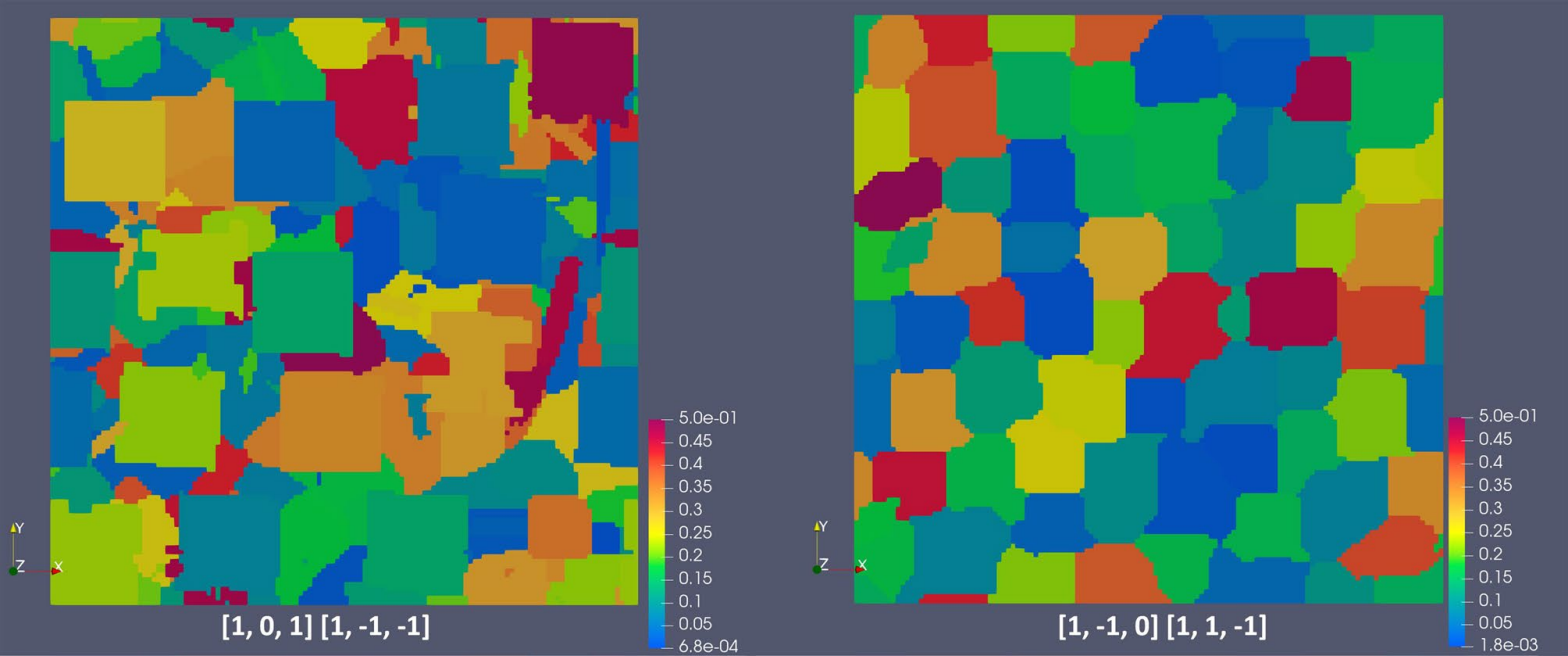
Color maps with largest Schmid factors for RVEs from Figs. 7, 8 and 9 with indicated slip systems.

Figures 9 and 10 compare the stress and strain distribution maps for IN738LC RVE from SEM/EBSD/X-Ray statistical data with parameters for CP from Table 3 and for hypothetically heat treated RVE with cuboidal grains shown in Fig. 8b. They display the crystal orientation maps in IPF colors, the X-component maps of Piola–Kirchhoff stress, the equivalent von Mises stress (based on the Cauchy stress tensor) map forecasting the yielding of the material for multi axial loads and the equivalent von Mises strain maps to visualize the total “shear” strain in the material. In addition, Fig. 11 depicts the color maps of two RVEs from Figs. 9 and 10 for slip systems with largest Schmid factors. As it can be seen from Figs. 9 and 10, the largest equivalent von Mises stress concentration is observed at grain boundaries with largest crystal orientation mismatch, though mapping could be complex due to three-dimensional morphology of the microstructures. The plastic deformations are also expected in grain colonies with easy active slip transfer.

From Figs. 9, 10 and 11, there is no clear correlation between stress/strain and Schmid factor values in polycrystal RVEs, i.e. the expected von Mises stress/strain localization in grains with softer crystallographic orientations (higher Schmid factor). However, the polycrystals generally require activation of two or more slip systems due to compatibility constraints unlike single crystal or bicrystals with arbitrarily oriented grains having generally one slip system activation^43^. As a result, the correlation with single slip system is not generally correct. From Figs. 8, 9, 10 and 11 and simulations with RVE having randomly-shaped non-columnar microstructure (not shown), it is evident that higher 0.2% proof stress is expected for LPBF manufactured IN738LC with columnar-type microstructure without irregularly-shaped precipitates which increase material yielding.

However, further investigations on other synthetic microstructures with LPBF are necessary for more detailed microstructure/property understanding and effect of the cracks.

## Conclusions

This work demonstrates the importance, route, and expected challenges for CP simulations of mechanical properties of as-build LPBF metal materials. The main results of present work can be listed as following:

1. To our best knowledge, the CP simulations with SSC predictions for LPBF-manufactured industrially-important IN738LC material were reported for the first time.
2. It was demonstrated that a very complex statistical copy of experimental sample can be reflected in its synthetic RVE with Dream3D for subsequent CP simulations. Several different morphology, texture, and spatial grain anisotropic distributions were introduced to our RVE in order to match the experimental EBSD data. Judging from listed literature on RVE reconstruction of additive manufactured materials, our realistic RVE has the most complex structure to date: the combination of cube octahedron and supper ellipsoid grain shapes with different grain size PDFs, spatial ODFs/RDFs as well as crystal ODFs which match the experimental statistical EBSD data. This demonstrates the possibility of modeling of even more complex microstructures which could be formed with AM methods.
3. The reasonable correspondence between experimental and simulated SSCs were achieved.
4. From fitted SSCs it can be concluded that all our experimental tensile tests of IN738LC material fabricated with LPBF method correspond to the strain hardening with linear isotropic slip hardening process and nonlinear strain rate sensitivity.
5. With our reconstructed RVE, the simulated tensile tests also demonstrate experimentally observed weak anisotropic behavior depending on tensions along and across of LPBF building directions. This suggested that major responsible factor was not columnar grain morphology, but rather their particular crystal orientation.

The remaining challenges and possible solutions for future studies are listed below:

1. For RVEs including cracks, our simulations did not converge. Therefore, if simulation time and PC resources are limited, then DAMASK Spectral Method can be used for samples without cracks due to their high phase contrast. Otherwise, FEM CP simulations are preferable or modified Spectral Methods should be used^35^. Regarding FEM for CP simulations, this method can handle non-smooth fields or discontinuities more accurately and robustly than spectral methods since it is based on piecewise polynomial functions as shape functions. So, it does not suffer from aliasing errors or Gibbs phenomenon and can achieve better convergence and accuracy for complex microstructures with cracks or interfaces. However, higher computational cost, much longer simulation times, mesh dependence, and difficulty in handling of large deformations are possible disadvantages of FEM compared to spectral method for CP simulations.
2. The manual parameter optimization in phenomenological model was also a challenge and generally should be replaced with an automated one. The etalon or reference SSCs should be measured or found in literature for reasonable parameter optimizations.

## Data Availability

The data and datasets used and analysed in current study are available from Dr. Makoto Watanabe on reasonable request. He can be contacted through the corresponding author.

## References

[1] Andani MT, Karamooz-Ravari MR, Mirzaeifar R, Ni J (2018). Micromechanics modeling of metallic alloys 3D printed by selective laser melting. Mater. Des..

[2] Somlo K (2022). Anisotropic yield surfaces of additively manufactured metals simulated with crystal plasticity. Eur. J. Mech. A Solids.

[3] Acar SS, Bulut O, Yalçinkaya T (2022). Crystal plasticity modeling of additively manufactured metallic microstructures, 2nd International workshop on plasticity, damage and fracture of engineering materials. Procedia Struct. Integr..

[4] Fischer T, Hitzler L, Werner E (2021). Morphological and crystallographic effects in the laser powder-bed fused stainless steel microstructure. Crystals.

[5] Van Nuland TFW, van Dommelen JAW, Geers MGD (2021). Microstructural modeling of anisotropic plasticity in large scale additively manufactured 316L stainless steel. Mech. Mater..

[6] Motaman SAH (2020). Optimal design for metal additive manufacturing: an integrated computational materials engineering (ICME) approach. The 2nd Asia-Pacific International conference on additive manufacturing (APICAM2019). JOM.

[7] Pauza J, Rollett A (2021). Simulation study of hatch spacing and layer thickness effects on microstructure in laser powder bed fusion additive manufacturing using a texture-aware solidification Potts model. JMEPEG.

[8] Cao M, Liu Y, Dunne FPE (2022). A crystal plasticity approach to understand fatigue response with respect to pores in additive manufactured aluminium alloy. Int. J. Fatigue.

[9] Pilgar CM, Fernandez AM, Lucarini S, Segurado J (2022). Effect of printing direction and thickness on the mechanical behavior of SLM fabricated Hastelloy-X. Int. J. Plast..

[10] Pinz M, Benzing JT, Pilchak A, Ghosh S (2022). A microstructure-based porous crystal plasticity FE model for additively manufactured Ti-6Al-4V alloys. Int. J. Plast..

[11] Azhari F (2022). A Comparison of statistically equivalent and realistic Microstructural representative volume elements for crystal plasticity models. Integr. Mater. Manuf. Innov..

[12] Motaman SAH, Roters F, Haase C (2020). Anisotropic polycrystal plasticity due to microstructural heterogeneity: A multi-scale experimental and numerical study on additively manufactured metallic materials. Acta Mater..

[13] Motaman SAH, Haase C (2021). The microstructural effects on the mechanical response of polycrystals: A comparative experimental-numerical study on conventionally and additively manufactured metallic materials. Int. J. Plast..

[14] Zhang W (2021). Very-high-cycle fatigue behavior of AlSi10Mg manufactured by selected laser melting: Crystal plasticity modelling. Int. J. Fatigue.

[15] Zhang J (2022). High-cycle and very-high-cycle fatigue lifetime prediction of additively manufactured AlSi10Mg via crystal plasticity finite element method. Int. J. Fatigue.

[16] Saha S, Kafka OL, Lu Y, Yu C, Liu WK (2021). Macroscale property prediction for additively manufactured IN625 from microstructure through advanced homogenization. Integr. Mater. Manuf. Innov..

[17] Groeber MA, Jackson MA (2014). DREAM.3D: A digital representation environment for the analysis of microstructure in 3D. Integr. Mater. Manuf. Innov..

[18] Roters F (2019). DAMASK—The Düsseldorf advanced material simulation kit for modelling multi-physics crystal plasticity, thermal, and damage phenomena from the single crystal up to the component scale. Comput. Mater. Sci..

[19] Nomoto S, Segawa M, Watanabe M (2021). Non- and quasi-equilibrium multi-phase field methods coupled with CALPHAD database for rapid-solidification microstructural evolution in laser powder bed additive manufacturing condition. Metals.

[20] http://dream3d.bluequartz.net/

[21] https://damask2.mpie.de/

[22] https://www.paraview.org/

[23] Bostanabad R (2020). Reconstruction of 3D microstructures from 2D images via transfer Learning. Comput. Aided Des..

[24] Bargmann S (2018). Generation of 3D representative volume elements for heterogeneous materials: A review. Prog. Mater. Sci..

[25] Sun Q, Jain MK (2022). Computational elastic analysis of AA7075-O using 3D-microstructrure-based-RVE with really-distributed particles. Int. J. Mech. Sci..

[26] MacSleyne JP, Simmons JP, Graef MD (2008). On the use of moment invariants for the automated analysis of 3D particle shapes. Model. Simul. Mater. Sci. Eng..

[27] Callahan PG, Groeber M, De Graef M (2016). Towards a quantitative comparison between experimental and synthetic grain structures. Acta Mater..

[28] Eisenlohr P, Diehl M, Lebensohn RA, Roters F (2013). A spectral method solution to crystal elasto-viscoplasticity at finite strains. Int. J. Plast..

[29] Sedighiani K (2020). An efficient and robust approach to determine material parameters of crystal plasticity constitutive laws from macro-scale stress–strain curves. Int. J. Plast..

[30] Bayerlein U, Socke HG (1992). Determination of single crystal elastic constants from ds- and dr-Ni-based superalloys by a new regression method between 20 °C and 1200 °C. Superalloys.

[31] Rabiei M (2020). Measurement modulus of elasticity related to the atomic density of planes in unit cell of crystal lattices. Materials.

[32] DAMASK — the Düsseldorf advanced material simulation kit, Spectral solver load definition, https://damask2.mpie.de/bin/view/Documentation/LoadDefinition (Accessed 1 September 2022) (2019).

[33] Maiti T, Eisenlohr P (2018). Fourier-based spectral method solution to finite strain crystal plasticity with free surfaces. Scr. Mater..

[34] Perevoshchikova N (2017). Optimisation of selective laser melting parameters for the Ni-based superalloy IN-738 LC using Doehlert’s design. Rapid Prototyp. J..

[35] Segurado J, Lebensohn RA, LLorca J (2018). Chapter one—computational homogenization of polycrystals. Adv. Appl. Mech..

[36] Diehl M (2010). A spectral method using fast Fourier transform to solve elastoviscoplastic mechanical boundary value problems. Diploma Thesis.

[37] Boyd JP (2001). in Chebyshev and Fourier spectral methods.

[38] Brisard S, Dormieux L (2010). FFT-based methods for the mechanics of composites: A general variational framework. Comput. Mater. Sci..

[39] Michel JC, Moulinec H, Suquet P (2001). A computational scheme for linear and non-linear composites with arbitrary phase contrast. Int. J. Numer. Methods Eng..

[40] Lucarini S, Dunne FPE, Martínez-Pañeda E (2023). An FFT-based crystal plasticity phase-field model for micromechanical fatigue cracking based on the stored energy density. Int. J. Fatigue.

[41] Weber G, Pinz M, Ghosh S (2020). Machine learning-aided parametrically homogenized crystal plasticity model (PHCPM) for single crystal Ni-Based superalloys. JOM.

[42] Aravindh NRR (2022). Micromechanical modeling of AlSi10Mg processed by laser-based additive manufacturing: from as-built to heat-treated microstructures. Materials.

[43] Roters F (2010). Overview of constitutive laws, kinematics, homogenization and multiscale methods in crystal plasticity finite-element modeling: Theory, experiments, application. Acta Mater..

